# Liver cell therapy: is this the end of the beginning?

**DOI:** 10.1007/s00018-017-2713-8

**Published:** 2017-11-27

**Authors:** Salamah M. Alwahsh, Hassan Rashidi, David C. Hay

**Affiliations:** MRC Centre for Regenerative Medicine, University of Edinburgh, 5 Little France Drive, Edinburgh, EH16 4UU UK

**Keywords:** Hepatocyte-like cells (HLCs), Co-culture, Cell tracking, Mesenchymal stem cells (MSCs), Cell sheet, Biomaterials, Extracellular matrix, Hepatic progenitor cells, Engraftment, Differentiation

## Abstract

The prevalence of liver diseases is increasing globally. Orthotopic liver transplantation is widely used to treat liver disease upon organ failure. The complexity of this procedure and finite numbers of healthy organ donors have prompted research into alternative therapeutic options to treat liver disease. This includes the transplantation of liver cells to promote regeneration. While successful, the routine supply of good quality human liver cells is limited. Therefore, renewable and scalable sources of these cells are sought. Liver progenitor and pluripotent stem cells offer potential cell sources that could be used clinically. This review discusses recent approaches in liver cell transplantation and requirements to improve the process, with the ultimate goal being efficient organ regeneration. We also discuss the potential off-target effects of cell-based therapies, and the advantages and drawbacks of current pre-clinical animal models used to study organ senescence, repopulation and regeneration.

## Introduction

Acute and chronic liver damage are significant causes of human ill health. The World Health Organization (WHO) estimated that 46% of global diseases and 59% of mortality are due to chronic diseases, with more than one million deaths in 2010 [1]. Thirty-five million individuals in the world die each year from chronic diseases and the numbers are increasing steadily [2]. Management of those patients with liver disease is complicated, but in most cases, the acute and end-stage liver diseases are treated by orthotopic liver transplantation (OLT). However, the shortage of ‘healthy’ donor organs results in a considerable disparity between the number of patients on the waiting lists and available organs. This results in patient mortality whilst on the waiting list for OLT [3].

There is a growing collection of therapeutic options that may benefit patients with acute or chronic liver disease including: the use of marginal grafts; defined as an organ with an increased risk for poor function or failure [4, 5], living donors, and cell-based therapies. Among the factors contributing to organ shortage crisis are steatotic organs, cost, cultural and psychological barriers to donation [6]. Marginal grafts also refer to the “expanded” donor and “extended criteria” donor [7], for example, elderly donors, split livers, steatotic livers, and donors with long ischaemia times. This increased use of marginal grafts has also been driven by data demonstrating that marginal grafts may be used with favorable outcomes [8]. In addition, due to a critical shortage of cadaveric organs for adults, the donor pool has been expanded to living donors. In most cases, this procedure requires the removing of the large right lobe of the liver from a donor [9].

Liver cell transplants attempt to provide organ support and promote liver regeneration. Recently, hepatocyte transplantation has been used to treat patients with liver failure [10, 11]. However, the use of fresh human hepatocytes has limitations which include: organ availability, limited cell proliferation, loss of function, and the risk for immune rejection [12]. New understanding of the mechanisms behind of liver regeneration and differentiation, including hepatic progenitor cells and pluripotent stem-derived liver cells, provides a renewable and genetically defined cell source for clinical trials in the future. The use of those cell types in two- and three-dimensional (2D and 3D) formats is being developed to generate tissue-like structures of clinical relevance.

Recent advances in genome editing have further aided the field, with the promise of disease-corrected cells for clinical treatment of hereditary diseases such as Crigler–Najjar syndrome, glycogen storage disease [13, 14] and hemophilia A (Factor VIII deficiency) [15]. Other potential candidates for liver cell therapy include familial hypercholesterolemia and Wilson’s disease [16] and advanced liver cirrhosis [17]. In addition to disease-causing mutations, promising applications of cell therapy have been evidenced in preclinical models of liver failure, such as acetaminophen-induced acute toxicity [18, 19].

Our review discusses the current progress in the field and ongoing research and development (R&D), with a focus on cell isolation, cell differentiation, optimal cell delivery and long-term performance in vivo.

## Current therapeutic options and on-going trials

Therapeutic options for the end-stage liver disease are successful but are extremely limited. Thus, the level of mortality whilst on the waiting is high as 20% [20]. To date, hepatic steatosis is a common clinical problem in developed countries, affecting up to 30% of donors for liver transplantation [21–23]. Due to the shortages of healthy liver donors, steatotic liver grafts have been clinically trialled. However, compared to normal livers, steatotic livers are particularly susceptible to ischemia/reperfusion (I/R) injury and oxidative stress, leading to poor outcome following surgery [5, 24–27]. Encouragingly, the blockade of CD47 (integrin associated protein, involved in apoptosis) with a specific antibody significantly reduced the extent of the I/R injury in donor steatotic liver allografts in rats, increasing survival of lean recipients, and represents a potential strategy for the use of fatty livers [28].

Numerous attempts have also been made to improve transplant outcome with healthy donor organs. Several methods of graft preservation have been developed, including simple cold storage, hypothermic machine perfusion, normothermic machine perfusion, and oxygen persufflation. A gradual rewarming of cold-preserved livers for 4 h by placement on normothermic extracorporeal liver perfusion (NELP) demonstrated beneficial effects in porcine livers. This included the reduction of the hepatocellular damage, reduced Kupffer cell activation and less cholangiocyte and sinusoidal endothelial cell dysfunction [29]. Despite the current progress, a significant unmet clinical need exists. This has prompted the development of alternative approaches to treat compromised liver function and disease [30].

## Regeneration of the normal liver

Liver parenchymal cells turn over slowly, but display high regenerative capacity, and are capable of restoring 70% tissue loss within a few weeks following injury [31]. The liver is composed of different cells types including hepatocytes, cholangiocytes, liver progenitor cells (LPCs, accounting for ~ 0.3–0.7% of healthy liver mass), hepatic stellate cells, Kupffer cells and endothelial cells. In uninjured livers, the source of newly generated hepatocytes has remained to be identified. Font-Burgada et al. have defined a distinct population of hepatocytes that exist in the periportal region of the uninjured mouse liver was so-called hybrid hepatocytes (HybHP), as these cells express several bile duct-enriched genes, including low amounts of Sox9 [31]. HybHPs are highly efficient in repairing of livers deficient in healthy hepatocytes [31]. Lineage-tracing results demonstrate that the biliary compartment may not play a role in normal liver homeostasis [32, 33]. Restoration of hepatocyte mass is mediated by the replication of remaining healthy hepatocytes (and cholangiocytes) in minor liver injury [34]. By lineage tracing in mice, a population of proliferating and self-renewing cells adjacent to the central vein in the liver lobule have been identified [33], while others showed there is no preferential proliferation across hepatocytes from all zones [35].

## Liver progenitor cell niche

The LPCs, known also as hepatic stem cells, are located at the level of Canals of Hering which are small branches of intrahepatic biliary trees [36]. The liver progenitor cells niche is rich in laminin which maintains their characteristics [37]. They exhibit bipotential plasticity [38] and are capable of regenerating both biliary and hepatic epithelia [39]. LPCs demonstrate proliferative capacity after acute and chronic liver damage in human livers [40–43]. However, the engraftment and repopulation efficacies of LPCs are considered to be lower compared to mature hepatocytes [13].

### Controversies in the role of the LPCs in liver regeneration

Controversial findings have been obtained using rodent and zebrafish models regarding the origin of cells that exhibit a proliferative capacity in injured livers [32]. Therefore, it is highly debated whether the cells adjacent to the portal tract [31, 43] or central vein [33] are responsible for liver regeneration. Understanding the LPCs contribution to hepatocyte generation is essential to better understand the mechanisms of liver regeneration and translating this to a favorable resolution of human liver injury [44, 45].

### Injured livers and the response of LPCs

Cells responsible for hepatocyte restoration in an injured liver remain not fully characterized. In a mouse model of liver injury, hepatocyte self-replication seems to provide practically all hepatocyte regeneration with minimal contribution from LPCs in the absence of senescence [46, 47]. When a considerable injury is incurred, resident LPCs are activated and expand from the periportal to the pericentral zone giving rise to reactive ductules. Reactive ductules, known as the ductular reaction, are strands of LPCs representing a population of cells with variable phenotypes [36]. They constitute a heterogeneous population of proliferating cells ranging from cells expressing stem cell markers with an immature phenotype, to more committed cells with an intermediate hepatobiliary phenotype [44, 48].

### Ductular reactions

For LPCs differentiation, Notch and Wnt are required, and their interaction is necessary for appropriate delineation of hepatocellular or biliary fates [49]. LPCs also express osteopontin and these cells are thought to emerge from bile ducts, capable of directly differentiating into hepatocytes [50]. Importantly, LPCs regenerate hepatocytes following chronic hepatocyte injury but not following biliary injury, demonstrate that the microenvironment is critical for HPC expansion and fate choice [36].

Ductal Lgr5^+^ stem cells can give rise to hepatocytes in vivo and in vitro [51]. These ductular reactions are important for biliary regeneration after cholestatic injury (by bile duct ligation) [52]. Rodrigo-Torres and colleagues found that the contribution of hepatocyte nuclear factor 1β (HNF1β^+^) biliary duct cells to liver regeneration was dependent on the liver injury model. HNF1β^+^ cells do not contribute to hepatocyte mass in the healthy liver, but after certain liver injury, they can differentiate into hepatocytes contributing to liver regeneration [44].

It has been reported that ductular cells spread from the portal tract in choline-deficient ethionine diet-induced hepatocellular injury in mice [53, 54]. During this insult, a massive hepatocyte loss is observed, where the remaining hepatocytes are unable to replicate. In this context, LPCs provide a hepatocyte-regenerating capacity to restore epithelial cell mass, architecture, and function [34, 43]. It still needs to be clarified in a variety of models whether hepatocytes can contribute to ductular cell populations. Evidence exists that hepatocytes can partially contribute to the ductular cell population [55]. Most notably, it was shown that when hepatocyte regeneration is impaired, ductular cells act as facultative stem cells to regenerate the liver parenchyma [56].

## Animal models to study liver repopulation following cell transplantation

The significance of the therapeutic benefits achieved in preclinical models, traditionally rodents and zebrafish, leads to new clinical interventions [32]. Several animal models have been developed to show “proof of principle” that transplanted hepatocytes are capable of replacing liver tissue and restoring liver function after engraftment in the recipient’s liver. A summary of various models used in studying liver regeneration and grafting of cells is discussed below.

### Genetically modified models

The first group of in vivo models is characterized by genetic modifications of the host liver that cause a severe liver injury providing transplanted hepatocytes with a strong growth/repopulation advantage. Sandgren et al. developed a transgenic mouse in which the overexpression of an albumin-urokinase-type plasminogen activator (Alb-uPA) fusion construct led to increased plasma uPA concentrations, resulting in a severe liver damage. Importantly, they observed that three or fewer transgene-deficient cells in the liver parenchyma were able to effectively reconstitute > 90% of the hepatic mass [57, 58].

The establishment of several immunodeficient animal models enables human hepatocyte transplantation. The severe combined immunodeficiency (SCID) [59] and recombinase 2 gene (*Rag2*) knockout [60] mice have spontaneous mutations in the *prkdc* locus or targeted mutation disrupting *Rag2* results in a lack of mature T and B cells, respectively. Whereas Beige [61] and perforin 1 gene (*pfp*) knockout [62] mice have mutations that lead to an impairment of natural killer (NK)-cell function. *Il2* (*γC*) knockout targeting mutation disrupts the Il2 receptor gamma chain gene. The knockout creates a lack of functional receptors for many cytokines leading to an impaired lymphocyte development and a lack of NK cells [63]. Non-obese diabetic (NOD) SCID gamma mice (NSG, NOD.CB17-*Prkdc* ^*scid*^/J) and NOG mice (NOD.Cg-Prkdc^*scid*^ Il2rg^*tm1Sug*^/Jic) are also excellent recipient mouse models for human cell engraftment [64].

 Inducible double knockout models have been established as well. Lu et al. have induced hepatocyte injury and senescence in adult mouse liver; the inducible AhCre Mdm2^flox/flox^ which is triggered by β-napthoflavone administration. Deletion of Mdm2 results in accumulation of p53 and induces p53-mediated cell death and senescence. This results in a rapid activation of LPCs throughout the liver, which in turn proliferate and differentiate into hepatocytes [43]. Fumarylacetoacetate hydrolase knockout (*Fah* ^−/−^) *and Rag2* ^−/−^ *Il2rg* ^−/−^ mice lack the B cells, T cells, and NK cells are completely immunodeficient. FAH is part of a subpathway involved in phenylalanine and tyrosine degradation, and its deficiency causes metabolic liver disease [65]. The advantage of this mouse strain is that liver injury can be controlled by 2-(2-nitro-4-trifluoro-methyl-benzoyl)-1, 3 cyclohexanedione (NTBC) administration that restricts toxic metabolite accumulation [66].

### Models of induction liver and biliary injury

Studying the performance and engraftment of transplanted human hepatocytes has been performed in various models of liver injury, as presented in Table 1. These models can be used to study liver regeneration, in the context of non-alcoholic steatohepatitis [67], fibrosis/cirrhosis [68], partial hepatectomy (PHx), or administration poisoning using carbon tetrachloride (CCl_4_) [32]. Whereas, the 3,5-diethoxycarbonyl-1,4-dihydrocollidine (DDC) diet has been used to induce biliary injury. Other protocols for preconditioning the liver by hepatic irradiation [69, 70], portal vein embolization, and surgical resection are effective for clinical trials.

**Table 1:** Induction of liver failure models

Treatment	Model	Term	Age (weeks)	Regenerative cells^a^	Graft	% Engraftment	References
Acetaminophen (paracetamol)	Mouse	Acute	4–6	VAL9 hESCs, hiPSC derived cells	1 × 10^6^ GFP-labeled VAL-Hep, intrasplenic	10.2, at day 30 post-Tx	[18, 44]
2-Acetylaminofluorine	Rat	Chronic	7–8	HPCs, WB-F344	–	–	[192]
Carbon tetrachloride	Mouse	Acute	ND	LPCs	–	–	[44]
Diethoxycarbonyl-1,4-dihydro-collidin (DDC)-diet	Mouse	Chronic	ND	HNF1β^+^	–	–	[44]
Choline-deficient ethionine-supplemented (CDE)-diet	Mouse	Chronic	ND	HNF1β^+^	–	–	[44]
Atherogenic + high fat diet^b^	Mouse	Chronic	8	HCs	1 × 10^5^ GFP-Tg ADSCs, intrasplenic	Detected ≤ 2 weeks in liver post-Tx	[67]
Tamoxifen	Mouse	Chronic	ND	GFP^+^HNF4^+^ CK19^−^ HCs. YFP^+^CK19^−^ HNF4α^+^ periportal HCs HybHP^c^	–	Tracked for 9 months	[31]
Diethylnitrosamine	Mouse	Chronic	6–9	hESC/iPSC-derived HCs	0.1–2 × 10^6^ hESC/iPSC-derived HLCs, i.v.	2–17% in the liver, 8 weeks post-Tx	[111]
Retrorsine + PHx	Rat	Acute	4	HCs	5 × 10^6^ HCs, intrasplenic	Detected ≤ 2 weeks in liver post-Tx	[39, 68]
	Mouse	Acute	ND	HCs, MSCs	2 × 10^6^ hu fetal HCs and/or hFLMSC	Co-Tx 81,000/mm^3^ liver	[72]
Monocrotaline	Rat	Acute	8–10	DPPIV^+^ HCs	1 × 10^7^ fresh DPPIV^+^ HCs, intrasplenic	≤ 1000 cells/50 consecutive liver lobules 3 months post Tx	[193]
Irradiation + PHx	Rat	Acute	7–8	HCs + fibroblasts	Co-Tx fibroblast + multilayered HCs sheets, s.c.	Detected 2 months post-Tx	[69]
HCV	Mouse	Chronic	5–8 months	hESCs- and hiPSCs-derived HLCs	4 × 10^6^ HLCs, intrasplenic	–	[194]
Ganciclovir	Mouse	Chronic	7–8	Endothelial	12 EGFP- or KO1-hiPSC-LB on the mesentery	–	[145]
Diphtheria toxin					3–4 × 10^6^ hu HCs under kidney capsule	–	
Thioacetamide	Mouse	Acute	7–8	ADMSC, HLCs, CK8^+^	5 × 10^6^ EGFP-labeled ADMSC, intrasplenic or i.v.	Detected ≤ 4 weeks in liver post-Tx	[126]
Phenobarbital + CCl_4_	Rat	Chronic	38	–	–	–	[67]
Galactosamine	Rat	Acute	8–10	–	8.8–10.5 × 10^6^ HC, i.p.	1 week post-Tx	[174]

*ADMSC* adipose-derived mesenchymal stem cells, *HC* hepatocytes, *HLC* hepatocyte-like cell, *HPCs* hepatic progenitor cells, *HybHP* hybrid hepatocytes, *hiPSC-LB* human induced pluripotent stem cells-liver buds, *s.c.* subcutaneously, *i.p.* intraperitoneal, *i.v.* intravenous, *Tx* transplantation, *EGFP* enhanced green fluorescent protein or human Kusabira-Orange (KO1) for live imaging [126], *hFLMSC* human fetal liver mesenchymal stem cells, *ND* or (–) non determined, *DPPIV* dipeptidyl peptidase IV

^a^The cell populations that are involved in liver regeneration per experiment

^b^Composed of cocoa butter, cholesterol, cholate, and corticotropin-releasing factor-1

^c^HybHP have an elevated Sox9 promoter activity and expression of other ductal markers were studied in *Sox9*-*Cre* ^*ERT*^ *;R26R* ^*tdTomato*^ mice

In other studies, rodents were treated by CCl_4_ with or without PHx and retrorsine; a cell cycle inhibitor, to block the proliferation of native hepatocytes. Subsequently, mice received freshly isolated β-galactosidase-labeled liver cells [71] or fetal liver-derived mesenchymal stem cells (MSCs) [72] to investigate their efficiencies in compensating the injured livers. Analogously, 5 × 10^5^ tail vein-injected multipotent MSCs improved liver regeneration and function in obese mice with hepatic steatosis after 70% PHx [73]. Interestingly, human umbilical cord-derived MSC transfusion has been reported to improve liver function in acute-on-chronic liver failure patients associated with HBV infection [74]. Likewise, the plant-derived pyrrolizidine alkaloid, monocrotaline, causes widespread injuries to hepatocytes, liver sinusoidal endothelial cells (LSECs), and Kupffer cells [75, 76]. Ingestion of 0.2% of ursodeoxycholic acid and cholic acids for 5 days caused cholestasis, apoptosis and liver injury in the bile salt export pump knockout (*Bsep* ^−/−^) mice [77]. After intrasplenic transplantation of freshly isolated wild-type hepatocytes, biliary total bile acids increased significantly after 1 week in recipient *Bsep* ^−/−^ mice.

A clinically relevant model, in which acute liver failure (ALF) has been induced in NOD/SCID young mice by 300 mg/kg acetaminophen (APAP) administration, has been developed. Three hours after APAP injection, intrasplenically transplanted GFP-labeled VAL9 hepatocytes (2 × 10^6^) were able to engraft and repopulate up to 20% of the liver, rescuing the mice after 2–8 weeks of the transplantation [18].

### Limitations of modeling liver disease in rodents

In animals, hepatocyte transplantation has been shown to correct enzymatic, receptor, or transport defects [16]. However, these positive results cannot be always translated through to successful clinical therapies. Hence, there is a need for more translational preclinical models to recapitulate the severity of liver injury seen in human disease [16]. Differences between animal models and clinical studies of liver regeneration involve signals and cellular sources that control liver regeneration [32]. Moreover, genetic diversity of rodents (inbred versus outbred) do not capture the wide diversity seen in patients. Furthermore, challenges, such as the route of cell administration to ensure optimal cell engraftment and function need to be further considered, as do the size of laboratory models.

## Potential cell sources to treat liver disease

Chronic scarring, fatty liver disease, prior chemotherapy and massive liver injury can all inhibit the normal program of liver regeneration and lead to liver failure [32]. Cell-based therapies provide a promising alternative to solid organ transplantation in patients with liver diseases [78, 79]. Cell therapy could also delay disease progression and facilitate a more aggressive resection of the liver in patients with hepatocellular carcinoma [16]. In addition, hepatocyte therapy has been successfully employed in humans to treat hepatic insufficiency or inborn metabolic disorders using primary human hepatocytes (PHH) [80]. Two billion viable hepatocytes were infused via portal vein into a glycogen storage disease type 1a patient, followed by immunosuppression regimens. Nine months post-transplant, the patient was able to eat a normal diet and could fast for 7 h without experiencing hypoglycaemia [81]. However, organ scarcity, allograft immune rejection, and difficult logistics of PHH use have driven researchers to explore alternative cell sources, including liver cell lines and pluripotent stem cells [82]. Below, we discuss the potential sources of hepatic cells that could be used in the clinic to treat liver diseases.

### Novel cell sources for transplantation

#### Pluripotent stem cells

Pluripotent stem cells (PSCs) display the ability to self-renew and retain pluripotency [83]. Human embryonic stem cells (hESCs) are one example and may serve as a renewable source of human tissue. More recently, Takahashi and Yamanaka generated induced pluripotent stem cells (iPSCs) from somatic cells using a combination of four reprogramming factors, including Oct4 (Octamer-binding transcription factor-4), Sox2 (Sex-determining region Y)-box 2, Klf4 (Kruppel Like Factor-4), and c-Myc [84, 85]. Therefore, in theory, PSCs could be used in combination or as an alternative to hESCs in various clinical or research settings, with the added benefit that iPSC-derived liver tissue can be immune matched [86–90].

#### PSCs-derived hepatic cells

PSCs have been shown to efficiently differentiate into hepatocyte-like cells (HLCs) [91]. Several groups have reported the generation of HLCs from PSCs through stepwise protocol by using various growth factors [92–94], or by combinational transduction of FOXA2 and HNF1α. These directed HLCs have many hepatocyte characteristics inducing cytochrome P450 enzyme activity, the ability to uptake LDL and Indocyanine green, store glycogen, and synthesize urea [95]. Although PSCs are promising cell sources for the mass production of HLCs, their limitations include incomplete gene expression, scale-up limitations, and heterogeneous culture [96]. With the consensus that PSC-derived HLCs are phenotypically and functionally more similar to fetal human hepatocytes [96, 97].

#### Improving of HLC differentiation and function

Advancements in cell culture matrices and media have led to significant improvement in HLC function and viability [91, 98]. Moreover, the generation of functional human ES-derived HLCs has been developed under chemically defined conditions which are compatible with good manufacturing practice (GMP) grade for clinical applications [91, 99]. Recently, Cameron et al. have improved hepatic specification and function by using fully defined recombinant laminin substrates (laminin 521 and 521/111 blend) as coating matrix [98, 100]. Laminin matrix was also used to improve the differentiation of HLCs from human bone marrow MSC (hBM-MSCs) [101]. These approaches should facilitate the development of clinical grade hepatocytes for transplantation.

Small molecules that can activate the mesenchymal–epithelial transition and induce the rapid proliferation have been also developed for improving the differentiation towards hepatic cells. Of these small molecules, A-83-01, an inhibitor of Smad signaling, inhibits TGFβ-induced epithelial–mesenchymal transition and CHIR99021, an inhibitor of glycogen synthase kinase 3β (Gsk-3β), enhances iHepgeneration by inducing rapid proliferation of somatic cells [102]. Siller et al. have devised a growth factor-free protocol that relies on small molecules to efficiently differentiate human PSCs toward a hepatic phenotype [103], however, their approach still relied on fetal bovine serum and Matrigel, limiting GMP-grade. In addition, *Hnf1α* supported by a cocktail of small molecules was sufficient to induce direct hepatic reprogramming from mouse fibroblasts. The induced hepatocyte-like cells (iHeps) by this technology represented functional hepatic cells [102].

In addition, to improve the function of ESCs-derived hepatic cells, human ESCs or iPSCs have been transduced with several transcription factors to promote their differentiation in vitro [95]. Adenovirus vector-mediated *Hnf4α* overexpression led to an upregulation of epithelial and mature hepatic markers, such as cytochrome P450 enzymes, and secretion of urea and albumin [95]. A multistage procedure including hepatocyte growth factor was used to differentiate hESCs directly into HLCs which exhibit mature hepatocyte morphology, and express albumin and HNF4α [91, 93, 94]. Further refinement and development of more sophisticated, a non-viral based, methodologies are still required to generate improved and phenotypically stable HLCs for downstream application [96, 104].

Transplantation of HLCs could also represent an alternative to OLT in ALF, late-stage liver disease such as cirrhosis, or in maintaining liver function in patients who do not meet the clinical eligibility for OLT [30]. Key advantages of replacing primary hepatocytes with HLCs are summarized in Table 2.

**Table 2:** Advantages of cell-based therapy

Factor	Cell transplant	Organ transplant
Cost	Less	More expensive
Complexity	Simple administration via intravascular catheters	Complex surgery
Availability	Large scale	Limited
Invasiveness	Minimal	Involve incision, open surgery
Occurrence	Could be provided repeatedly/multiple recipients	Usually one/patient

#### Stem cell-derived hepatic cells: post-transplant challenges

Before cell transplantation for treating liver disease, cells should fulfill numerous criteria, including safety, reproducibility, xeno-free, and long-term function (Table 3). ES-derived cells are allogeneic and, therefore immunogenic, increasing the risk for allograft rejection and necessitating immunosuppression [105]. It has been shown that HLCs led to a tumor after transplantation. Payne et al. reported that human ES-derived hepatocytes recipient mice developed large splenic and liver tumors that contained endodermal and mesodermal cell types 3 months post-transplantation [106]. Despite current shortfalls, PSC-derived HLCs provide a unique opportunity to study the mechanisms involved in human hepatocyte differentiation and liver function in more detail [93]. Encouragingly, HLCs generated from hESCs or human iPSCs have been shown to model human liver disease ‘in a dish’ [107] and to accurately predict and modulate human drug-induced hepatotoxicity [91, 108–110]. Optimistically, when hESC-derived HLCs were injected into immunocompromised mice, after CCl_4_-induced liver injury, human serum albumin was detected for 3 months post-transplantation [106]. Additionally, it has been reported that iPSC-derived HLCs successfully repopulated the liver tissue and secreted human-specific liver proteins in the blood of mice with a liver cirrhosis [111].

**Table 3:** Criteria of in vitro generated cells that they should meet to be used in cell therapy for liver diseases

Criteria	hPSC [98]	hESCs, iPSCs [103, 195]	hESC-derived HLCs [106]	ADHLPCs [112, 113]	Autologous iPSCs [87]	hESC-derived HLCs [196]
GMP-grade	√	×	×	√	×	×
Xeno-free	√	×	×	×	×	×
Immunogenicity^a^	NT	NT	NT	√^b^	√	NT
Tumorogenicity	NT	NT	√	√^c^	×	NT
Scalability	√	√	√	√	√	√
Resistance to cryopreservation	NT	NT	NT	√	NT	√
Long-term efficacy	NT	NT	√	√	√	√
Display mature hepatocyte functions	√	√	√	√	NT	√

*NT* not tested, *ND* not determined, *GMP* good manufacturing practice, *ADHLPCs* adult-derived human liver progenitor cells

^a^For in vivo use, immunocompromised (mouse) models were used

^b^Tested in Ref. [112]

^c^Tested in Ref. [113]

In addition, adult-derived human liver progenitor cells (ADHLPCs) have been tested for the degree of immunogenicity when they were co-cultured with allogeneic human adult peripheral blood mononuclear cells (PBMCs) [112] and tested for oncogenicity in 5-week-old Balb-c nude mice for 24 weeks [113], Table 3. Sana et al. reported that ADHLPCs were associated with a low immunogenic profile in vitro [112]. Tumorigenicity, phenotypic and genetic stability, and differentiation potential of ADHLPCs have been studied in vitro and in a xenograft assay. These cells, however, after a prolonged culture displayed cytogenetic instability [113].

### Induced hepatocyte (iHep) transdifferentiation in vivo

Direct induction of somatic cells into induced hepatocyte (iHep) that closely resemble hepatocytes has been achieved by viral transduction and expression of various transcription factors. Combinations of *Hnf4α* and *Foxa1*, *Foxa2* or *Foxa3* or *Gata4*, *Hnf1a* and *Foxa3* have been used to convert mouse embryonic or adult fibroblasts into iHep [114, 115]. More recently, human iHeps have been generated from fibroblasts by overexpression of FOXA3, HNF1α, and HNF4α. Upon transplantation into *Fah* ^−/−^ mice with concanavalin-A-induced acute liver failure, iHeps restored the liver function and prolong survival, demonstrating successful lineage conversion of non-hepatic human cells into hepatocytes [65]. Moreover, stellate cell transdifferentiation has been successfully carried out in vivo, in the context of liver fibrosis, providing proof of concept [116]. Direct cell lineage conversion through reprogramming is a promising field of research for different applications in regenerative medicine and personalized disease modeling.

#### Mesenchymal stem cells (MSCs)

Numerous studies have reported therapeutic effects of transplanted MSCs on hepatic fibrosis, cirrhosis, and other liver diseases, which may be related to the differentiation of MSCs into functional hepatocytes [117, 120]. MSCs are a group of pluripotent stem cells with self-renewal and multi-directional differentiation potential derived from mesoderm, which are widely distributed in various tissues, such as bone marrow, umbilical cord, and adipose tissues. MSCs have been transplanted alone or with HLCs to treat injured liver (Table 4). HLCs have been generated also from placenta-derived human amniotic epithelial cells (hAEC) and placed in barium alginate microcapsules to prevent immune cell-mediated rejection post-transplantation [118]. The differentiated HLCs performed key functions of hepatocytes during 7 days in culture without losing their viability. An improvement of liver function has been achieved in patients having end-stage liver disease (liver cirrhosis, hepatitis B virus (HBV), HCV, and alcoholic) after autologous MSC injection [117, 119, 120]. However, the improvements in survival rate in cirrhotic patients using autologous bone marrow-derived MSC transplantation through peripheral vein was not significant, based on randomized controlled trial [121].

**Table 4:** Various sources of stem cells

Stem cells	References
hESCs	Carpentier 2014 [194], Cameron 2015 [98]
hiPSCs	Yu 2007 [88], Beers 2015 [89], Wang 2017 [100]
Bone marrow-derived MSCs	Mohamadnejad [121]
Autologous mesenchymal stem cells (AMSCs)	Kharaziha 2009 [119], Amer 2011 [124]
Mesenchymal stem cells (MSCs)	Xu 2017 [125]
Human adipose-derived stem cells (ADSCs)	Harn 2012 [122], Seki 2013 [67]
Human Amnion epithelial cells	Vaghjiani 2014 [118]
Umbilical cord-derived mesenchymal stem cell (UC-MSC)	Li 2016 [123]
Hepatic progenitor cells (HPCs)	Sacho-Bru 2012 [42], Lu 2015 [43]

#### Intrahepatic and extrahepatic MSC transplantation

Intrahepatic and extrahepatic MSCs transplantation approaches have been trialled and their contribution in liver regeneration has been studied. Human adipose-derived stem cells (ADSCs) differentiate into albumin-secreting HLCs after 1 week of intrahepatic transplantation in thioacetamide (TAA)-induced rat model of chronic liver damage [122]. Patients with HBV related acute-on-chronic liver failure (HBV-ACLF) received plasma exchange, entecavir and a single transplantation of umbilical cord-derived MSC (UC-MSC) transplantation (100 × 10^6^ cell suspension via the hepatic artery) compared to another group which received only plasma exchange and entecavir. Notably, an improvement in liver function tests and higher cumulative survival rate at 24 months have been observed in the patient groups who were transplanted with UC-MSC [123].

In addition, extrahepatic transplantation of MSCs is thought to play an important role in liver repopulation. The outcomes of intrasplenic or intrahepatic autologous bone marrow MSC-derived hepatocytes transplantation have been evaluated in patients with end-stage liver cell disease. The results showed significant improvement in ascites, edema, serum albumin, and performance status, and amelioration of fatigue scale, demonstrating the safety and short-term efficacy of autologous bone marrow-derived MSC injection in treating liver failure [124]. Amer et al. found no significant difference between the outcomes observed from the group which received cells directly into the liver (intrahepatic) and indirectly (intrasplenic) [124]. MSC-seeded silk fibroin matrices were placed onto the liver surface of mice with ALF showed an obvious therapeutic ability for injured liver function [125]. The effects of different routes of adipose-derived MSC (ADMSC) transplantation on the restoration of liver functions in acute mouse liver failure were assessed. Mice were injected with enhanced GFP-labeled ADMSCs by intrasplenic or intravenous (tail vein) routes. Transplantation via tail vein provided a significant survival benefit compared to intrasplenic cell administration [126]. Furthermore, the transplanted cells were well integrated into injured livers and produced albumin and cytokeratin-8 in both groups. Deng et al. concluded that direct intravenous ADMSCs transplantation is an effective treatment for ALF rather than intrasplenic transplantation [126].

MSCs have positive effects during regeneration such inhibiting apoptosis in hepatocytes and Kupffer cells, secreting of various bioactive molecules to promoting liver regeneration and reducing inflammation [117]. In addition, MSCs contribute in the restoration of the liver parenchymal tissue by hepatocytes or/and LPCs, and also they could contribute in the wound healing after injury in terms of angiopoiesis by liver sinusoidal endothelial cells or/and sinusoidal endothelial progenitor cells [127]. These findings highlight the importance of different sources and options for MSCs in the treatment of liver disease.

## Could bioengineering approaches be exploited in improving cell-based therapy?

Bioengineering approaches may provide alternative approaches to improve cell engraftment and/or function and to control ESC and iPSCs differentiation into distinct cell and tissue types. While both “top–down” and “bottom–up” approaches have been used for biofabrication, the emergence of additive manufacturing or three dimensional (3D) bioprinting has brought manufacturing of functional tissue one-step closer to reality. In combination with defined culture media and suitable growth factors, researchers have made significant progress in this space, although at present HLCs are less functional than their PHH counterparts. In the following sections, we discuss the new bioengineering systems to improve HLC or organoid culture, activity and their long-term function in vitro and in vivo.

### Extracellular matrix: 2D and 3D cultures

The extracellular matrix (ECM) plays an important role in cell organization and function. Phenotypic instability of primary hepatocytes and HLCs in vitro is a major obstacle to the widespread application [128]. 2D systems have proved to be invaluable for studying basic cell biology and are facile and cost-effective to use. However, these systems are associated with limitations such as remodeling of the internal cytoskeleton and limited cell–cell contact [129] and cell–ECM interaction provide a suboptimal environment to cells. To overcome these problems, various strategies have been explored including co-culture with non-parenchymal liver cells and development of 3D platforms to better mimic in vivo microenvironment [82, 128]. The 3D systems can be categorized into scaffold-based and scaffold-free systems. In scaffold-based systems, synthetic or natural materials are employed to provide support to the cultured cells, while in scaffold-free platforms self-aggregation of cells is a major driving force to generate 3D structures.

Scaffold-based 3D culture systems allow the production and organization of cells in vitro in a controllable and reproducible manner. Several synthetic polymers, such as a poly lactic co-glycolic acid (PLGA) and natural materials, such as collagen, have been evaluated for in vitro differentiation and maintenance of HLCs [130]. PSC-derived HLCs have also been cultured on polyurethane-coated scaffolds which supported cell phenotype and performance in vitro [131]. More recently, modulation of hepatic function was investigated following co-culture of rat bone marrow-derived MSCs and freshly isolated hepatocytes on a PLGA scaffold. Cell-seeded PLGA scaffolds have been transplanted into the abdominal cavity of mice. The result suggested that the best in vitro and in vivo performance can be achieved using a 1:5 ratio of MSC to hepatocytes [132].

Due to the complexity of ECM and its tissue-specific properties, the role of the decellularized liver has been evaluated by several groups. Improved cell function was achieved following culture of iPSCs-derived HLCs on liver-derived ECM compared to poly-l-lactic acid (PLLA) scaffold coated with collagen or Matrigel [133]. Mazza et al. have also developed a protocol for complete decellularization of a whole human liver to form an extracellular matrix scaffold with a preserved architecture a preserved architecture [134]. Biocompatibility was demonstrated by either omental or subcutaneous xenotransplantation of liver scaffold cubes into immunocompetent mice for 3 weeks, resulting in absent foreign body responses or local signs of inflammation.

To circumvent some of the limitations presented by scaffold-based methodologies, several methods have been developed to generate scaffold-free microtissues. While culture on ultra-low adherent surface and molds are among the most common approaches, hanging drop, spinner culture, rotating wall vessels and microfluidic-based methodologies have also been used. Recently, FP001, a low-acyl gellan gum polymer, was used to form of HepaRG [135] 3D spheroids and maintenance of the culture without the requirement for stirring [136]. Nanopillar plate technology has also been applied to control the configuration of spheroids and to generate more mature HLCs [110].

## Organoid cell culture

Stem cell-derived organoids are 3D human micro-tissues generated in vitro [137]. The development of methodology was pioneered by the Clevers’ lab and culture system for several organs has been developed including intestine [138], stomach [139], pancreas [140] and the liver [141, 142]. Long-term maintenance and expansion of liver organoids in undefined conditions has been achieved from liver biopsy to form functional hepatic cells both in vitro and in vivo [51]. A more recent protocol describes how to grow adult mouse and human liver and pancreas organoids in a gel-based ECM and defined medium in vitro [143]. Epithelial organoids recapitulate multiple aspects of real organs, making them promising models of organ function to model and treat human disease [137, 144].

### Co-culture

In line with previous studies, co-culture of human iPSC-derived hepatic endoderm with umbilical vein endothelial cells and MSCs resulted in a generation of improved liver buds. Following mesenteric transplantation, the liver buds improved the survival of mice following ganciclovir-induced liver failure [145]. A recent study has shown that the co-culture strategy could improve mouse survival than those treated with one cell type [132]. The transplantation of organoid-PLGA scaffold has significantly rescued the damaged liver in mice and showed a lower immunogenic response level compared to single MSC-PLGA or Hep-PLGA scaffold treatments [132]. Furthermore, aggregation into organoid-like structures has been tested using PHHs and stem cell-derived HLCs with MSC co-culture, and yielded promising results both in terms of cell function and engraftment [146].

Co-cultures of different cell types have been also used to form transplantable organoids. Asai et al. have identified paracrine signals that regulate the differentiation of human hepatocytes. For this purpose, they established a liver organoid using human iPSCs, MSCs, and human umbilical vein endothelial cells. The cells have self-assembled into 3D organoids, resulting in improved hepatocyte differentiation [129]. Multicellular liver organoid units composed of heterogeneous cell populations that contain human adult stem and progenitor cells, hepatocytes, bile ducts and vascular structures, hepatic stellate cells, and endothelial cells have been transplanted under the abdominal skin in immunodeficient mice with a liver failure [147]. The transplanted organoids provided a functional support up to 4 weeks post-transplantation [147]. While these studies are very encouraging, it is not yet feasible to transplant organoids via blood vessels, due to their large size, making routine deployment difficult.

## Modular biofabrication

Construction of large tissue fragments through an assembly of smaller modules is known as modular biofabrication. Such a “bottom–up” approach is an alternative to conventional “top–down” methodology of seeding a large porous scaffold. Various techniques have been developed including cell sheet [148], microfabrication of cell-laden hydrogels [149], and self-assembled aggregation [150]. Intraportal or intrasplenic hepatocyte transplantation can create issues, such as cell death, embolism, portal vein thrombosis and hypertension [151, 152]. To get around this, cell sheet-based tissue engineering approaches have been developed to deliver a large number of cells to the desired location while preserving cell to cell contact and ECM, and reducing the loss of transplanted cells [64, 153–156]. Early attempts used temperature responsive surfaces to manufacture cell sheets from PHHs. Efficient engraftment and long-term stability for longer than 200 days were observed following subcutaneous implantation [157]. More recently, Kim et al. developed a methodology to generate endothelial–hepatocyte hybrid sheets [154]. Bile canaliculi networks were formed among the hepatocytes in the hybrid cell sheet. Albumin secretion level was preserved for 4 weeks in the hybrid Hep-EC sheet, which was in contrast to hepatocytes cultured in a monolayer [154].

A similar approach was used to manufacture hybrid cell sheets consisting of human fibroblasts PHH cell sheets to improve neoangiogenesis following subcutaneous implantation [64]. Most recently, a fetal liver mesothelial cell sheet was developed to prevent postoperative adhesion in a mouse model of PHx. Interestingly, secreted paracrine growth factors enhanced proliferation of hepatocytes after PHx indicating the potential to improve regeneration [158]. Additionally, human iPSC-derived HLC sheets were implanted directly onto the surface of the liver after performing PHx. Considerable cell engraftment and liver-specific protein production were observed in cell sheet transplanted mice compared to the control group receiving a similar number of cells through intrasplenic infusion. In addition, cell sheet recipient mice were rescued from CCl_4_-induced lethality, and provided better therapeutic support than intrasplenic infusion [12].

## Improving hepatocyte isolation and preservation

Hepatocytes are isolated from cadaveric donors, primarily in the form of resected segments recovered after hepatectomy. Several parameters are critical in this process, affecting both the quality and yield of hepatocyte prepartion. This includes the donor’s medical history, preservation conditions, and the isolation procedure [159]. Generally, parenchymal and non-parenchymal cells are isolated from the liver by collagenase perfusion with a low-speed centrifugation, density gradient centrifugation, and magnetic-activated cell sorting. This yields cell populations of high purity and quality [160].

Although freshly isolated primary human hepatocytes (PHHs) are the gold standard for various cell-based modeling and for in vivo transplant, they are limited by scarcity and ‘shelf life’. Therefore, current strategies have focused on improving cryopreservation media and protocols to preserve hepatocyte phenotype. Being able to cryopreserve human hepatocytes will allow for the repeated use of the same pre-characterized hepatocyte lots in different studies [161], permitting lab to lab comparisons [161].

Cryopreservation refers to the processes required to maintain the health and function of biologics outside the body, and to ensure a return to function post-resuscitation. Cellular damage occurs due to the freezing process [162] which is reduced when hepatocytes are suspended in human plasma [163]. Hepatocyte pre-incubation with medium supplements such as, sugars, insulin, reduced glutathione (GSH) and *N*-acetyl-l-cysteine prior to cryopreservation has also been shown improve cell recoveries after thawing [162]. The cryoprotectants used in freezing medium include, glycerol, dimethyl sulfoxide, polyvinylpyrrolidone and polyethylene glycol [162]. Trehalose has also been used in combination with 10% DMSO to stabilize the plasma membrane [164]. Post thawing, 3D approaches have been developed to maintain the long-term function of PHHs [161]. Recently, Nguyen et al. developed a bioprinting approach to building 3D liver tissue culture in a 24-well Transwell^®^ plate using PHHs, human hepatic stellate cells, and human umbilical vein endothelial cells [165].

## Routes of cell delivery and associated challenges

In liver cell therapy, studies have defined the critical components for the optimal repopulation by transplanted cells. This includes their isolation, characterisation, and storage prior to their engraftment in the liver (or extrahepatic sites) [16]. This requires an ideal route of transplantation that assures maximal cells delivery with the best engraftment. In addition, transplanted cells must survive over defined period of time and proliferate to impart therapeutic benefit. This may require preconditioning of recipients before or after cell transplantation and development of suitable regimens to control for allograft rejection [16].

**Fig. 1:**
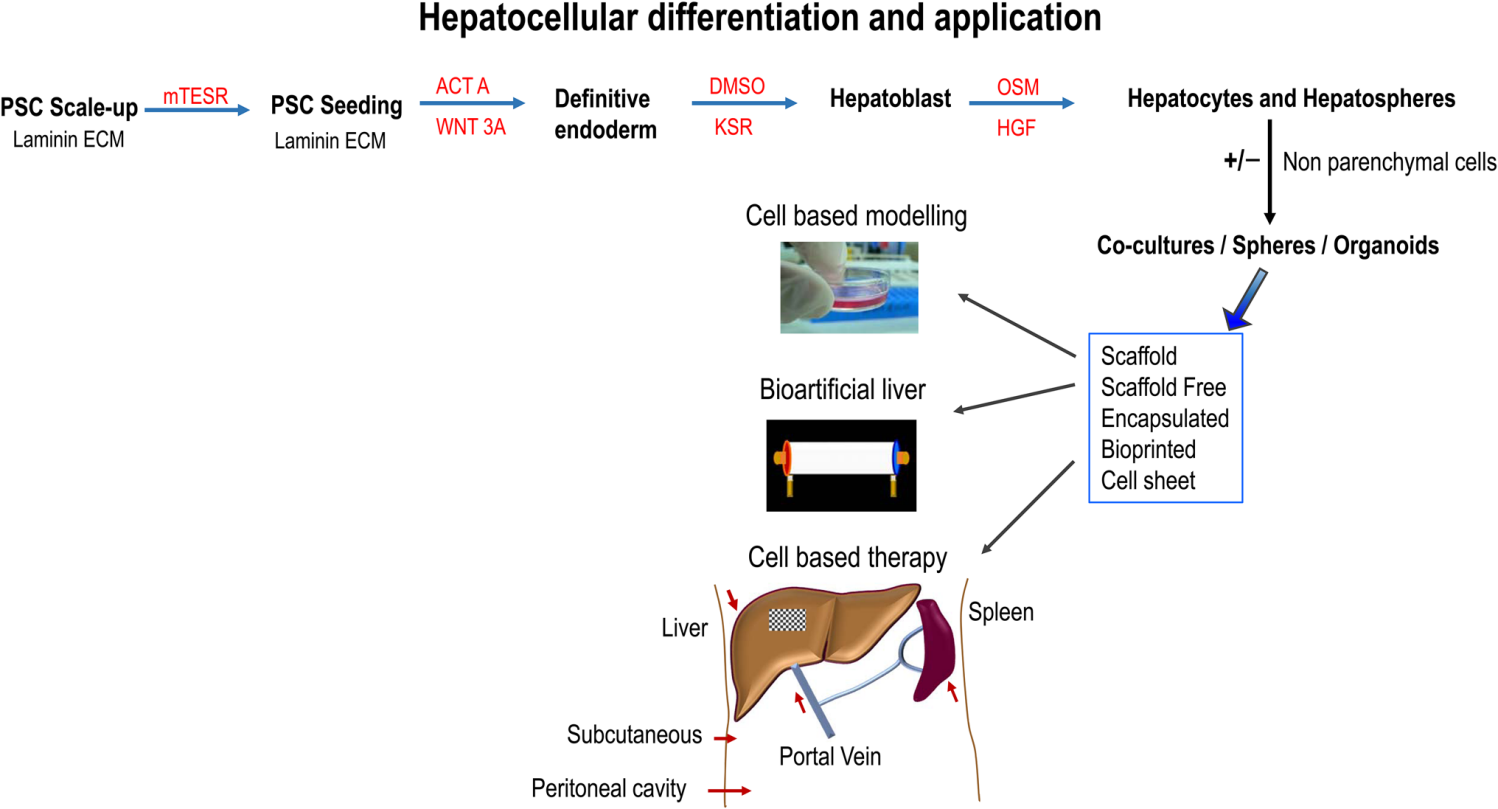
Directed differentiation of pluripotent stem cells (PSCs) and their potential applications. PSCs were maintained on laminin extracellular matrix (ECM) and differentiated toward hepatic tissue using a four-stage process employing Activin A (ACTA), Wnt3a, and using differentiation medium (80% knockout DMEM (KO-DMEM), 20% knockout serum replacement (KSR), GlutaMAX, non-essential amino acids, β-mercaptoethanol, 1% Dimethyl sulfoxide (DMSO), and penicillin/streptomycin), and HepatoZYME maturation medium supplemented with Oncostatin M (OSM) and human hepatocyte growth factor (HGF). Following differentiation and tissue engineering, monolayer, co-culture, sphere and organoids could be applied in the future to model human biology, generate artificial liver devices, and used as cell-based therapies in vivo. The liver is shown in brown, the spleen in reddish-brown, and the liver bandage as a patch on the liver. Arrows (red) point to the site of cell delivery

LPCs, PHH, and human iPS-HLCs have been transplanted into the rodents with liver injury, by intrasplenic or portal venous infusion [166, 167]. A suspension of BM-derived HLCs (0.5 × 10^6^–2 × 10^8^) has been also injected intravenously, intrasplenically or intrahepatically with ultrasonographic guidance [124]. The most common sites of ectopic hepatocyte transplantation in patients with major hepatectomy or liver cirrhosis are the spleen. The spleen can be accessed by direct injection into the splenic artery through a catheter inserted through the femoral artery [168]. Because of a high mortality rate-associated intrasplenic injection in NSG mice, human PSC-derived HLCs [111] or primary MSCs [73] were transplanted via tail vein injection. Other potential routes of cell administration are depicted in Fig. 1 and those include: subcutaneous cell implantation and immune isolation, transplanting human HLCs into immunocompetent mice following co-aggregation with stromal cells and encapsulation in a biocompatible hydrogel [169]. Alginate has also been employed to encapsulate the cells prior to implantation in the abdominal cavity [170]. The structure of formed microbeads is porous allowing for efficient mass transfer and protection from the immune system [171].

### Cell tracking

Tracking cells during regenerative cytotherapy is crucial for monitoring their engraftment, safety, and efficacy. A reliable, clinically applicable cell-tracking agent would be a powerful tool to study cell biodistribution, viability, performance, and differentiation. Superparamagnetic iron oxide nanoparticles (SPIONs) have been used to label macrophages for MRI-based cell tracking in vivo [172]. Similarly, magnetic labeling of primary and stem cell-derived pig hepatocytes has been employed for MRI-based cell tracking [173]. In addition, PLGA encapsulated magnetic nano- and micro-particles and photoconvertible near-infrared lipophilic cell membrane dyes have also been employed [174, 175].

Long-term in vivo monitoring of ADHLPCs has been performed to determine differences in biodistribution following intrasplenic and intrahepatic transplantation in immunodeficient/beige mice. In this experiment [176], ADHLPCs were transduced with a lentiviral vector expressing a triple fusion reporter comprising renilla luciferase, monomeric red fluorescent protein, and truncated HSV-1 thymidine kinase and were monitored by bioluminescence imaging, positron emission tomography, and contrast-enhanced computed tomography. It has been concluded that ADHLPCs could be monitored by bioluminescence imaging for up to 4 weeks with widespread biodistribution following intrasplenic injection [176]. These techniques and others such as bioluminescence and fluorescence have also aided the study of cell biodistribution and engraftment post-transplantation.

Clinically, cell labeling with ^111^In-oxine is a safe and suitable method for tracking the biodistribution of transplanted cells. A short-term biodistribution of ADHLPCs has been assessed over 5 days post portal vein infusion in a patient with glycogen storage disease type 1A. Defresne et al. labeled ADHLPCs with ^111^-Indium and single-photon emission computed tomography used for whole body imaging. No signal was detected in extrahepatic organs, confirming retention of the transplanted cells [14]. A patient suffered from hemophilia A has been injected over 50 days with 1.21 × 10^9^ ^111^In-DTPA radiolabelled ADHLPCs via the humeral vein. Biodistribution analysis revealed an initial temporary entrapment of the cells in the lungs, followed by homing to the liver and to a joint afflicted with hemarthrosis [15]. In vivo tracking of ^111^In-oxine labeled MSCs following intravenous infusion in patients with advanced cirrhosis revealed an accumulation of radioactivity in the lungs at first, and then it gradually increased in the liver and spleen in all patients within 10 days [17].

### Long-term function and off-target effects

Cell delivery is not the only concern when designing cell-based therapies. It is also important to monitor whether or not cells proliferate appropriately following engraftment [177]. Although several groups have administered human iPS-HLCs intrasplenically or intraperitoneally [111, 178], their engraftment efficiencies were limited. Using conventional cell administration methods, it is quite difficult to control for efficient engraftment and avoid off-target engraftment in other organs [12, 179]. Therefore, in addition to improving cell phenotype, preconditioning the liver niche may aid transplanted cells to engraft, function and persist [39]. Such approaches include PHx, portal embolization, liver irradiation, and repeated cell infusion [180]. Bile acids have been reported to accelerate liver regeneration [32, 181] as have growth factors [182] and the thyroid hormone T_3_ [183]. Several studies also demonstrate a key role of adiponectin during tissue regeneration [184, 185].

## Cell modeling and cell utility

Freshly isolated primary hepatocytes, cryopreserved hepatocytes, immortalized cancer cell lines, liver tissue slices and animal models broadly categorize the liver models available to study the human pathophysiology of the liver [186]. More recently, PSCs have been used to model human biology successfully recapitulating key features of the cellular pathology seen in liver disease [187]. Recent transcriptomic analysis of human NAFLD, cirrhosis, HCC and hepatitis B virus-infected tissue was compared to cultured cells, demonstrated a strong similarity between cell modeles and human disease [188].

In addition to genetic diseases, drug-induced liver injury is a complex problem which accounts for approximately 65% of ALF cases in the United States [189]. This demonstrates the requirement for a step change in current cell-based safety systems. There have been reports where human PSC-derived HLCs are capable of modeling drug-induced liver injury [95, 190]. Most recently, hepatoblasts, derived from pluripotent stem cells, have been employed to better understand in utero exposure to maternal smoking [191].

## Conclusions

There have been important advances in human liver regenerative medicine. This includes the development of scalable cell sources that offer potential to overcome the shortages of donor organs and hepatocyte instability, and challenges associated with the immune system. Despite those advances, stem cell-derived liver cells require further development before they are suitable for the clinic. On-going efforts are exploring numerous avenues to improve cell function, engraftment and stability in vitro and in vivo, offering an exciting future for the field.

## Abbreviations

ALF: Acute liver failure
HLCs: Hepatocyte-like cells
hESCs: Human embryonic stem cells
HybHP: Hybrid hepatocytes
iHeps: Induced hepatocytes
iPSCs: Induced pluripotent stem cells
NK: Natural killer cells
PHx: Partial hepatectomy
PHH: Primary human hepatocytes
PSCs: Pluripotent stem cells
2D: Two-dimensional
3D: Three-dimensional
LPCs: Liver progenitor cells
R&D: Research and development
OLT: Orthotopic transplantation
